# Clinical Trials of Non-Coding RNAs as Diagnostic and Therapeutic Biomarkers for Central Nervous System Injuries

**DOI:** 10.2174/1570159X21666221128090025

**Published:** 2023-09-01

**Authors:** Huiqing Wang, Qiang Wang, Xiao Xiao, Xiaolei Luo, Linbo Gao

**Affiliations:** 1 Medical Simulation Centre, West China Second University Hospital, Sichuan University, Chengdu, Sichuan, 610041, P.R. China;; 2 Laboratory of Molecular Translational Medicine, Center for Translational Medicine, Key Laboratory of Birth Defects and Related Diseases of Women and Children, Sichuan University, Ministry of Education, NHC Key Laboratory of Chronobiology, Sichuan University, West China Second University Hospital, Sichuan University, Chengdu, Sichuan, 610041, P.R. China;; 3 Department of Obstetrics and Gynecology, West China Second University Hospital of Sichuan University and the Key Laboratory of Birth Defects and Related Diseases of Women and Children, Sichuan University, Ministry of Education, Chengdu, P.R. China

## 1 INTRODUCTION

Central nervous system (CNS) injuries, including brain injury and spinal cord injury (SCI), often cause irreversible neurological damage and high medical care expenditure. In 2016, the worldwide age-standardized incidence rates of traumatic brain injury (TBI) and SCI were 369 and 13 per 100 000, respectively, with an estimated 27.08 million new cases of TBI and 0.93 million new cases of SCI [1]. While a diagnosis of severe TBI is generally uncomplicated, mild TBI (mTBI), especially mild closed head injury, is more challenging to detect. Non-coding RNAs (ncRNAs) have previously been considered as potential noninvasive diagnostic biomarkers or drug targets for several human diseases [2, 3]. In TBI and SCI, a series of ncRNAs, such as microRNAs (miRNAs), long-coding RNAs (lncRNAs), small nucleolar RNA (snoRNA), and wiRNA have been evaluated for their potential use as diagnostic and therapeutic biomarkers [4-11]. In this review, we summarize current advances in the application of ncRNAs as diagnostic and prognostic markers of CNS injuries based on data from clinical trials. The potential roles of ncRNAs in axon regeneration after CNS injuries are introduced. Additionally, the challenges and current directions of clinical studies are also discussed.

## 2 CLINICAL TRIALS OF ncRNAs AS BIOMARKERS IN THE DIAGNOSIS AND PROGNOSIS OF CNS INJURIES

The ClinicalTrials.gov database (https://clinicaltrials.gov/) currently lists a total of 49 clinical trials evaluating the potential role of ncRNAs as diagnostic and therapeutic biomarkers for CNS injuries (Table **1**). Among these trials, 20 focus on stroke, five on subarachnoid hemorrhage, nine on TBI, five on Parkinson’s disease (PD), five on epilepsy, three on amyotrophic lateral sclerosis (ALS), and two on SCI. As of July 20, 2022, 21 of the studies are still recruiting participants, 13 have been completed, and 3 were terminated. The estimated sample sizes used in the clinical trials exhibited large variations, ranging from 5 to 1,620. Only 12 studies were clinical phase trials, the majority (66.7%) being clinical phase II.

Previously, a large number of miRNAs have been reported to have diagnostic, monitoring, and management potentials in stroke, such as miR-155, miR-30a, miR-126, let-7b, miR-107, miR-128b, miR-153, miR-16, miR-335, miRNA-221-3p, miR-125a-5p, miR-125b-5p, miR-143-3p, miRNA-382-5p, and miRNA-4271 [4-9]. However, the clinical utility of these miRNAs remains inconclusive, given the lack of verified findings in large-scale prospective trials. To date, a total of 20 clinical trials have been carried out to investigate the expression patterns and evaluate the diagnostic or predictive value of ncRNAs in patients with stroke. Only two prospective observational studies (NCT04175691 and NCT04230785) published data on ncRNAs as biomarkers for the diagnosis and prognosis of stroke. Using four independent sets, a lncRNA-based combination index including three lncRNAs (linc-DHFRL1-4, SNHG15, and linc-FAM98A-3) was established. The combination index showed an area under curve (AUC) value of more than 0.84, indicating that a combination of these three lncRNAs might distinguish ischemic stroke patients from healthy controls [12]. In addition to these ncRNAs, miR-124-3p, miR-125b-5p, miR-192-5p, and miR-206 were reported to be promising indicators of stroke severity and functional outcome in acute ischemic stroke patients receiving thrombolysis (Table **2**) [13, 14].

Delayed cerebral infarction (DCI) is a common complication of subarachnoid hemorrhage (SAH) with high morbidity and mortality. Multiple studies have been conducted to determine the extent to which miRNA expression can serve as a diagnostic tool for SAH. Results from two case-control studies (NCT03344744 and NCT01791257) showed that miR-21, miR-221, miR-4532, miR-4463, miR-1290, and miR-4793 may differentiate SAH patients with DCI from those without DCI [15, 16]. When combining miR-4532, miR-4463, miR-1290, and miR-4793, the AUC reached 0.82 (Table **2**) [16]. However, the study had a relatively small sample size (20 SAH patients with DCI and 20 SAH patients without DCI), and the results should be confirmed in larger replication studies.

Given that the diagnosis of mild TBI (mTBI) remains a critical challenge due to subtle signs and syndromes and the absence of reliable and objective biomarkers, several studies have been conducted to assess the value of ncRNAs in the diagnosis and outcome prediction of mTBI. For example, a prospective observational study (NCT02639923) found that plasma levels of miR-92a and miR-16 were significantly increased in mTBI patients within the first 24 hours post-injury, with AUC values of 0.78 and 0.82 for the two miRNAs, respectively [10]. In addition to plasma-induced ncRNAs, salivary ncRNAs may alternatively be used to discriminate mTBI from healthy controls. In 2016, a multicenter clinical trial (NCT02901821) was carried out to assess the ability of ncRNAs to predict symptom duration and character following mTBI. The study recruited 538 individuals (251 mTBI patients and 287 controls) who were divided into testing and training groups. Random forest was performed to create an mTBI‐predictive algorithm based on datasets of single ncRNA and ncRNA ratios. The age of participants and seven ncRNA ratios were included in the ncRNA model, and the AUC for differentiating mTBI from controls was 0.86 in the training set and 0.82 in the testing set (95% confidence interval (CI): 0.82‐0.90). Using an expanded model combining clinical variables (age of participants, symptom severity, symptom burden) and the expression of miR-4510, miR-27a-5p, miR-1246, and wiRNA_2048, the AUC for differentiating mTBI from controls was 0.93 (95% CI: 0.89‐0.97) [17]. Furthermore, machine learning techniques were used to predict the status of persistent post-concussion symptoms. The prognostic algorithm included 16 ncRNAs and the age of participants, and reached an AUC of 0.83 (95% CI: 0.81‐0.85). When identifying symptom recovery at 21 days post-injury, the best ncRNA model consisted of miR-12136, miR-200a-5p, miR-203a-5p, miR-423-5p, wiRNA_3506, wiRNA_9363, wiRNA_9447, wiRNA_10135, RNU1-1, RNVU1-17, SNORD18B, and age of participants (Table **2**) [18].

Previous work has also investigated whether circulating ncRNAs can be used as potential biomarkers for SCI [11]. However, no validated markers have been identified so far. Two clinical trials (NCT04379011 and NCT03965299) are currently being conducted with the aim of establishing a set of circulating miRNA-based biomarkers for clinical outcome and intervention after SCI.

TBI has been established as a predisposing factor for a variety of neurodegenerative diseases, including ALS, PD, and epilepsy [19]. Numerous studies have presented ncRNAs as potential biomarkers for the diagnosis and therapeutics of these diseases [20-24]. For instance, serum miR-146a, miR-106b, miR-301a, miR-194-5p, let-7d-5p, miR-130a-3p, and miR-15a-5p may represent potential biomarkers for epilepsy [22, 23]. Similarly, miR-27a, miR-133a, miR-133b, miR-206, and miR-181 might be used as non-invasive circulating biomarkers for the diagnosis and prognosis of ALS (Table **2**) [20, 21, 24]. Moreover, miR-181, widely expressed in neurons, is sufficient to induce dopaminergic neuronal loss by suppressing a set of genes related to synaptic transmission, neurite outgrowth, and mitochondrial respiration [24, 25]. Five clinical trials (NCT03466723, NCT02672943, NCT02283073, NCT04651140, and NCT03918616) were previously performed to determine non-invasive biomarkers for PD; however, no ncRNAs originating from these clinical trials have been reported.

## 3 ncRNAs IN AXON REGENERATION AND NEURITE OUTGROWTH AFTER CNS INJURY

**Fig. (1):**
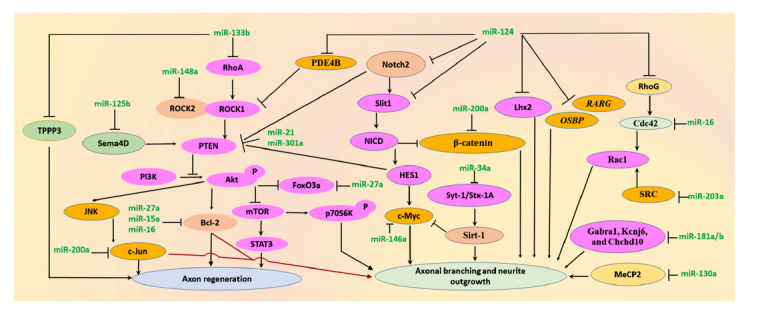
The potential roles and interactive networks of ncRNAs in neurite outgrowth and axon regeneration following CNS injury.

Despite the limited regenerative capacity of mature neurons following CNS injury, several methods have been developed over the past decades by manipulating the CNS environment to induce axonal regeneration and neurite outgrowth, including controlling miRNA expression [26-28]. miR-124 is a crucial regulator of CNS regeneration and exhibits altered expression in CNS injury [28-31]. Axonal branching and neurite outgrowth were observed following miR-124 over-expression, which decreases cell division cycle 42 (Cdc42) protein and promotes the subcellular localization of Rac family small GTPase 1 (Rac1) by targeting ras homolog family member G (*RhoG*) [32, 33]. Since a single miRNA has multiple target genes, miR-124 can also target LIM homeobox protein 2 (*Lhx2*), phosphodiesterase 4B (*PDE4B*), Rho associated coiled-coil containing protein kinase 1 (*ROCK1*), oxysterol-binding protein (*OSBP*), and retinoic acid receptor gamma (*RARG*), activate the PI3K/Akt signaling pathway, and suppress mTOR signaling activity, thereby inducing neurite elongation [34-38]. In an inflammatory microenvironment, miR-124 abrogates TNF-α-mediated inhibition of neurite outgrowth [39]. A knock-down of miR-124 disrupts planarian brain regeneration by either modulating *notch-2* or targeting slit guidance ligand 1 (*slit-1*), which encodes an axon guidance protein (Fig. **1**) [30].

miR-133b is another important determinant in axon outgrowth, albeit with different roles in different tissues. In an adult zebrafish SCI model, miR-133b down-regulation impairs locomotor recovery and decreases regeneration of axons from brainstem neurons by reducing the levels of Ras homolog gene family member A (RhoA) protein expression [40]. In rat and mice models of CNS injury, an exogenous injection of miR-133b improves functional recovery and neurite outgrowth through a reduction in RhoA, xylosyltransferase 1 (Xylt1), ephrin receptor A7 (Epha7), and purinergic receptor P2X ligand-gated ion channel 4 (P2RX4), and phosphorylated activation of mitogen-activated protein kinase 1/3 (ERK1/2), signal transducer and activator of transcription 3 (Stat3), and cAMP responsive element binding protein 1 (Creb) [41-43]. In a PD cell model, miR-133b over-expression ameliorates axon degeneration *via* targeting *RhoA* and up-regulating phosphorylated Akt [44]. In zebrafish single Mauthner-cells, inhibition of miR-133b expression promotes axonal regeneration by modulating tubulin polymerization-promoting protein family member 3 (Fig. **1**) [45].

In addition to miR-124 and miR-133b, other miRNAs also play key roles in neurite outgrowth and axon regeneration after CNS injury [46-49]. For example, miR-21, miR-34a, miR-181a/b, miR-146a, and miR-130a modulate neurite outgrowth both *in vivo* and *in vitro via* targeting multiple genes, such as phosphatase and tensin homolog (*Pten)*, synaptotagmin-1 (*Syt-1*), syntaxin-1A (*Stx-1A*), gamma-aminobutyric acid type A receptor subunit alpha1 (*Gabra1*), potassium inwardly-rectifying channel subfamily J member 6 (*Kcnj6*), coiled-coil-helix-coiled-coil-helix domain containing 10 (*Chchd10*), and methyl-CpG binding protein 2 (*MeCP2)* [25, 46, 50-52]. After SCI, miR-21 inhibition in astrocytes can increase axon density [47] and the precise post-injury level of miR-125b is essential for creating a regeneration-permissive environment [53]. Specifically, miR-125b expression must be tightly controlled to promote axon regeneration by targeting semaphorin 4D (*Sema4D*). *Sema4D* stimulates PTEN activity and induces growth cone collapse in hippocampal neurons [53, 54]. During spinal cord regeneration, miR-200a up-regulation is required in glial and progenitor cells, suppressing c-Jun and β-catenin expression, blocking AP-1^cFos/cJun^ formation, and eventually repairs the missing spinal cord tissue (Fig. **1**) [48, 49].

## CONCLUSION

Over the past two decades, the tissue-specific and differential expression features of ncRNAs have been studied extensively, and ncRNAs have emerged as highly potential targets for the diagnosis and treatment of CNS injuries. For example, the combination of miR-4510, miR-27a-5p, miR-1246, and wiRNA_2048 expression as a disease biomarker was found to have more than 90% accuracy in detecting mTBI [17], while the combination of linc-DHFRL1-4, SNHG15, and linc-FAM98A-3 expression had more than 80% accuracy in detecting stroke [12]. Despite these promising results, however, there are still some challenges that must be overcome before ncRNAs may be effectively implemented in clinical practice: (1) hundreds or thousands of ncRNAs have been identified to date, and the ncRNAs evaluated in different clinical trials are of diverse types. But there is still no widely accepted set of ncRNAs that are considered to be most suitable for clinical application; (2) most clinical trials to date have evaluated the diagnostic and prognostic value of ncRNAs in CNS injuries, but no study has yet assessed the therapeutic potential of ncRNAs as drug targets; (3) intracerebral drug delivery presents several challenges which further hamper clinical implementation. Exosomal ncRNAs carried by novel biomedical materials or intranasal delivery may provide new opportunities for the treatment of CNS injuries.

Until now, more than half of the clinical trials registered in the ClinicalTrials.gov database are ongoing. After these studies have been completed, more evidence will be provided, which might open new avenues for the diagnosis and prognosis of CNS injuries. Moreover, the exploration of novel CNS therapeutic drugs targeting ncRNAs will be of great importance in future clinical trials.

## Figures and Tables

**Table 1:** Clinical trial of non-coding RNAs in CNS injuries.

**Condition or Disease**	**Observational Model**	**Estimated Number of Participants**	**Status**	**NCT Number**	**Phase**	**Sponsor**
Stroke	Cohort	30	Completed (ASSD: August 2016; ASCD: February 2017; LUD: February 2017)	NCT02838589	2	Herlev-Gentofte Hospital, Denmark
Stroke	Cohort	20	Completed (ASSD: November 2016; ASCD: August 2017; LUD: August 2017)	NCT02801032	2	Herlev-Gentofte Hospital, Denmark
Ischemic stroke	Case-control	45	Completed (ASSD: July 2020; ASCD: July 2021; LUD: February 2022)	NCT04266639	-	Aarhus University Hospital, Denmark
Acute ischemic stroke	Case-control	1503	Completed (ASSD: March 2013; ASCD: March 2019; LUD: April 2019)	NCT01677208	4	China Academy of Chinese Medical Sciences, China
Ischemic stroke	Case-only	259	Completed (ASSD: December 2016; ASCD: October 2021; LUD: January 2022)	NCT02937077	-	Oslo University Hospital, Norway
Acute ischemic stroke	Case-control	46	Completed (ASSD: June 2014; ASCD: July 2020; LUD: July 2020)	NCT02176395	4	China Academy of Chinese Medical Sciences, China
Acute ischemic stroke	Cohort	300	Recruiting (ASSD: April 2019; ESCD: March 2023; LUD: August 2021)	NCT03892226	-	Charite University, Germany
Acute ischemic stroke	Case-control	500	Recruiting (ASSD: November 2019; ESCD: December 2023; LUD: April 2022)	NCT04175691	-	Nanjing First Hospital, Nanjing Medical University, China
Acute ischemic stroke	Cohort	300	Recruiting (ASSD: March 2020; ESCD: March 2025; LUD: April 2022)	NCT04230785	-	Nanjing First Hospital, Nanjing Medical University, China
Ischemic stroke	Case-control	30	Recruiting (ASSD: August 2016; ESCD: May 2022; LUD: November 2021)	NCT02829502	2	Herlev-Gentofte Hospital, Denmark
Stroke	Cohort	100	Recruiting (ASSD: June 2021; ESCD: January 2023; LUD: November 2021)	NCT04766645	-	Fondazione Don Carlo Gnocchi Onlus, Italy
Stroke	Case-control	120	Recruiting (ASSD: April 2021; ESCD: February 2024; LUD: November 2021)	NCT04382963	-	University of Wisconsin, United States
Stroke	Cohort	1620	Recruiting (ASSD: July 2020; ESCD: June 2035; LUD: November 2020)	NCT04399200	-	• University Hospital Grenoble, France
Stroke	Case-only	134	Recruiting (ESSD: August 2020; ESCD: March 2025; LUD: June 2020)	NCT04323501	-	Universita di Verona, Italy
Acute stroke	Case-control	1500	Recruiting (ASSD: April 2018; ESCD: December 2024; LUD: February 2022)	NCT03481777	-	Aarhus University Hospital, Denmark
Ischemic stroke	Cohort	600	Unknown (ASSD: June 2018; ESCD: December 2021; LUD: July 2018)	NCT03577093	-	Capital Medical University, China
Ischemic stroke	Cohort	120	Unknown (ASSD: August 2017; ESCD: December 2021; LUD: June 2019)	NCT03994003	-	Ospedale Civico, Lugano, Switzerland
Ischemic stroke	Case-only	100	Not yet recruiting (ESSD: May 2022; ESCD: December 2029; LUD: March 2022)	NCT05173896	2	Herlev-Gentofte Hospital, Denmark
Stroke	Case-control	280	Not yet recruiting (ESSD: May 2022; ESCD: January 2025; LUD: April 2022)	NCT05323916	-	Charles University, Czech Republic
Acute ischemic stroke	Cohort	45	Suspended (ASSD: August 2018; ESCD: August 2023; LUD: October 2021)	NCT03905434	-	Virginia Commonwealth University, United States
Subarachnoid hemorrhage	Case-control	70	Completed (ASSD: November 2014; ASCD: May 2015; LUD: May 2015)	NCT02320539	-	• Rigshospitalet Copenhagen, Denmark
Subarachnoid hemorrhage	Case-control	360	Recruiting (ASSD: November 2017; ESCD: December 2023; LUD: March 2022)	NCT03344744	-	Chinese University of Hong Kong, China
Subarachnoid hemorrhage	Case-control	50	Completed (ASSD: February 2013; ASCD: January 2014; LUD: April 2014)	NCT01791257	-	Bispebjerg Hospital, Denmark
Subarachnoid hemorrhage	Cohort	50	Completed (ASSD: September 2010; ASCD: October 2019; LUD: June 2021)	NCT02389634	-	St. Joseph's Hospital and Medical Center, Phoenix, United States
Subarachnoid hemorrhage	Cohort	198	Completed (ASSD: February 2012; ASCD: August 2017; LUD: March 2018)	NCT01670838	-	Kuopio University Hospital, Switzerland
Neonatal encephalopathy	Case-control	200	Enrolling by invitation (ASSD: August 2020; ESCD: September 2022; LUD: March 2021)	NCT04816331	-	University of Dublin, Trinity College, Ireland
TBI	Cohort	300	Recruiting (ASSD: January 2022; ESCD: November 2022; LUD: March 2022)	NCT05279599	-	Tang-Du Hospital, Fourth Military Medical University, China
TBI	Case-control	132	Recruiting (ASSD: January 2018; ESCD: July 2021; LUD: June 2021)	NCT01048138	3	Federal University of São Paulo, Brazil
mTBI	Cohort	150	Unknown (ASSD: January 2016; ASCD: July 2017; LUD: March 2017)	NCT02639923	-	• Medical University of Vienna, Austria
mTBI	Case-control	700	Recruiting (ASSD: January 2016; ESCD: September 2025; LUD: September 2021)	NCT02901821	-	Penn State Milton S. Hershey Medical Center, United States
mTBI	Cohort	32	Terminated (ASSD: September 2014; ASCD: June 2016; LUD: February 2018)	NCT02100150	2	Neuren Pharmaceuticals Limited, United States
Brain concussion	Cohort	750	Recruiting (ASSD: January 2021; ESCD: January 2026; LUD: September 2021)	NCT04582682	-	Milton S. Hershey Medical Center, United States
Brain concussion	Cohort	103	Active not recruiting (ASSD: November 2018; ESCD: August 2021; LUD: April 2021)	NCT03844282	-	University of Cambridge, United Kingdom
Brain concussion	Case-control	50	Terminated (ASSD: February 2017; ESCD: September 2020; LUD: January 2022)	NCT02969824	-	University of Toronto, Canada
Spinal cord injury	Cohort	20	Recruiting (ASSD: February 2021; ESCD: March 2023; LUD: February 2022)	NCT04379011	1 and 2	University of Minnesota, United States
Spinal cord injury	Cohort	114	Recruiting (ASSD: June 2019; ESCD: June 2024; LUD: July 2021)	NCT03965299	-	University of Zurich, Switzerland
ALS	Cohort	5	Terminated (ASSD: June, 2014; ESCD: October 2015; LUD: November 2018)	NCT01992029	-	University Hospital, Bordeaux, France
ALS	Cohort	36	Active not recruiting (ASSD: March 2021; ESCD: May 2023; LUD: April 2022)	NCT04840823	1 and 2	McGill University, Canada
ALS	Case-control	60	Recruiting (ASSD: December 2017; ESCD: December 2024; LUD: May 2022)	NCT03088839	-	Neuromed IRCCS, Italy
PD	Case-control	1000	Active not recruiting (ASSD: February 2019; ESCD: June 2022; LUD: May 2022)	NCT03466723	-	Neuromed IRCCS, Italy
PD	Case-control	60	Active not recruiting (ASSD: January 2016; ESCD: August 2017; LUD: February 2016)	NCT02672943	-	Chang Gung Memorial Hospital, Taiwan, China
PD	Case-control	410	Active not recruiting (ASSD: November 2014; ESCD: December 2019; LUD: July 2018)	NCT02283073	-	Bio Shai Ltd.
PD	Cohort	30	Enrolling by invitation (ASSD: October 2020; ESCD: October 2021; LUD: January 2021)	NCT04651140	-	The First Hospital of Jilin University, China
PD	Case-control	50	Completed (ASSD: February 2017; ASCD: March 2019; LUD: August 2019)	NCT03918616	-	University of Pisa, Italy
Post-traumatic Epilepsy	Cohort	132	Recruiting (ASSD: January 2018; ESCD: July 2021; LUD: June 2021)	NCT01048138	3	Federal University of São Paulo, Brazil
Epilepsy	Case-control	75	Completed (ASSD: March 2018; ASCD: March 2022; LUD: April 2022)	NCT03419000	-	Hospices Civils de Lyon, France
Epilepsy	Case-control	60	Completed (ASSD: August 2016; ASCD: August 2018; LUD: September 2018)	NCT02359188	1 and 2	Philipps University Marburg Medical Center, Germany
Epilepsy	Cohort	87	Recruiting (ASSD: December 2018; ESCD: November 2023; LUD: April 2022)	NCT04259125	-	University of California, United States
Epilepsy	Cohort	550	Recruiting (ASSD: February 2022; ESCD: February 2030; LUD: July 2022)	NCT05450822	-	Gitte Moos Knudsen, Denmark

**Abbreviations:** CNS, central nervous system; NCT, national clinical trial; ASSD, actual study start date; ASCD, actual study completion date; LUD, last update date; ESCD, estimated study completion date; ESSD, estimated study start date; TBI, traumatic brain injury; mTBI, mild traumatic brain injury; ALS, amyotrophic lateral sclerosis; PD, Parkinson’s disease.

**Table 2:** Diagnostic and prognostic roles of non-coding RNAs in CNS injury.

**Condition or Disease**	**Non-coding RNAs**	**Sample Type**	**AUC ** **(95% CI)**	**Sensitivity (%)**	**Specificity (%)**	**Targets**	**References**
Ischemic stroke	linc-DHFRL1-4, SNHG15, and linc-FAM98A-3	Mononuclear cells	0.84 (0.75-0.94)	81	72	-	[12]
Acute ischemic stroke	miR-124-3p, miR-125b-5p, and miR-192-5p	Plasma	0.80 (0.61-0.89)	88	65	miR-124: *RhoG, Lhx2, PDE4B, ROCK1, OSBP,* and *RARG;* miR-125b: *Sema4D*	[14]
Subarachnoid hemorrhage	miR-4532, miR-4463, miR-1290, and miR-4793	Serum	0.82 (0.69-0.96)	-	-	-	[16]
Severe TBI	miR-16	Plasma	0.89	-	-	*Cdc42, Cdc23, Bcl-2*, and *Cyclin-D1*	[10]
	miR-92a	Plasma	0.82	-	-	*Itgα5* and *KLF4*	[10]
	miR-765	Plasma	0.86	-	-	*BCL2L13*	[10]
	miR-16, miR-92a, and miR-765	Plasma	1.00	100	100	-	[10]
Mild TBI	miR-16	Serum	0.82	-	-	*Cdc42, Cdc23, Bcl-2*, and *Cyclin-D1*	[10]
	miR-92a	Serum	0.78	-	-	*Itgα5* and *KLF4*	[10]
	Age, miR-34a-5p/SNORD57, miR-34a-5p/SNORD104, miR-4510/SNORD59A, chronic headache/miR-27a-5p, miR-34a-5p/miR-192-5p, miR-192-5p/SNORD2, and SNORD57/SNORD75	Saliva	0.86 (0.82-0.90)	-	-	miR-34a: *Syt-1*, *Stx-1A*, and *Sirt1*; miR-27a: *Bcl-2* and *FoxO3a*	[17]
	Age, symptom severity, symptom burden, miR-4510, miR-27a-5p, miR-1246, and wiRNA_2048	Saliva	0.93 (0.89-0.97)	-	-	miR-27a: *Bcl-2* and *FoxO3a*	[17]
	Age, wiRNA_48, miR-486-5p, miR-1246, wiRNA_147, wiRNA_1500, wiRNA_9924, miR-92b-3p, wiRNA_7971, miR-203a-5p, SNORD81, wiRNA_9447, miR-148a-5p, wiRNA_1385, wiRNA_7876, miR-100-5p, and miR-148-3p	Saliva	0.83 (0.81-0.85)	81	73	miR-92b: *NOX4*; miR-203a: *SRC*; miR-148a: *Rock2* and *KLF6*	[18]
mTBI recovery	Age, miR-12136, miR-200a-5p, miR-203a-5p, miR-423-5p, wiRNA_3506, wiRNA_9363, wiRNA_9447, wiRNA_10135, RNU1-1, RNVU1-17, and SNORD18B	Saliva	0.86 (0.83-0.89)	-	-	miR-200a: *c-Jun*	[18]
ALS	miR-27a, miR-133a, miR-133b, and miR-206	Serum	-	-	-	miR-133b: *RhoA* and *TPPP3*	[21]
	miR-181	Plasma	-	-	-	*Gabra1, Kcnj6,* and *Chchd10*	[24]
Epilepsy	miR-146a	Serum	0.77 (0.68-0.82) or 0.78	82	65	*STAT1* and *c-Myc*	[22, 23]
	miR-106b	Serum	0.79 (0.69-0.82) or 0.88 (0.84-0.93)	80	81	*ACSL4*	[22, 23]
	miR-301a	Serum	0.67 (0.65-0.72)	-	-	*Pten*	[22]
	miR-194-5p	Serum	0.69 (0.66-0.73) or 0.81	72	75	*Runx3*, *TRAF6*, and *Bach1*	[22, 23]
	miR-146a/miR-106b	Serum	0.89 (0.79-0.93)	-	-	miR-146a: *STAT1* and *c-Myc;* miR-106b: *ACSL4*	[22]
	let-7d-5p	Serum	0.79	84	62	-	[23]
Epilepsy	miR-130a-3p	Serum	0.78	79	68	*MeCP2*	[23]
	miR-15a-5p	Serum	0.84	80	73	*AKT3, IL-10RA*, *Claudin-5, Bcl-2,* and *BDNF*	[23]

**Abbreviations:** CNS, central nervous system; AUC, area under curve; CI, confidence interval; TBI, traumatic brain injury; mTBI, mild traumatic brain injury; ALS, amyotrophic lateral sclerosis; PD, Parkinson’s disease.

## LIST OF ABBREVIATIONS

ALS: Amyotrophic Lateral Sclerosis
AUC: Area Under Curve
CNS: Central Nervous System
DCI: Delayed Cerebral Infarction
lncRNAs: Long-coding RNAs
miRNAs: MicroRNAs
ncRNAs: Non-coding RNAs
PD: Parkinson’s Disease
SAH: Subarachnoid Hemorrhage
SCI: Spinal Cord Injury
snoRNA: Nucleolar RNA
TBI: Traumatic Brain Injury
